# Amiloride-Sensitive Sodium Channels and Pulmonary Edema

**DOI:** 10.1155/2011/830320

**Published:** 2010-12-29

**Authors:** Mike Althaus, Wolfgang G. Clauss, Martin Fronius

**Affiliations:** Institute of Animal Physiology, Justus-Liebig University of Giessen, Wartweg 95, 35392 Giessen, Germany

## Abstract

The development of pulmonary edema can be considered as a combination of alveolar flooding via increased fluid filtration, impaired alveolar-capillary barrier integrity, and disturbed resolution due to decreased alveolar fluid clearance. An important mechanism regulating alveolar fluid clearance is sodium transport across the alveolar epithelium. Transepithelial sodium transport is largely dependent on the activity of sodium channels in alveolar epithelial cells. This paper describes how sodium channels contribute to alveolar fluid clearance under physiological conditions and how deregulation of sodium channel activity might contribute to the pathogenesis of lung diseases associated with pulmonary edema. Furthermore, sodium channels as putative molecular targets for the treatment of pulmonary edema are discussed.

## 1. Introduction

According to Fick's law, the anatomy of the human lung permits optimal gas exchange due to a large surface area and a thin diffusion barrier. The large surface area is generated by the division of airways into smaller gas exchange units (alveoli). Alveoli consist of two cell types, alveolar type 1 (AT1) and type 2 (AT2) cells. AT1 cells are large, flat cells that build the bulk of the alveolar surface. In contrast, AT2 cells are smaller cuboidal cells, which are active secretory cells and are responsible for the secretion of surface active proteins and lipids, which are referred to as surfactant. Both cell types form tight junctions and thereby build a polar organised epithelium with an apical, “air-faced”, and a basolateral, “blood-faced,” side. At the basolateral side, a thin basal lamina separates the alveolar epithelium from the small interstitium and the capillaries of the lung. For effective gas exchange to take place, O_2_ and CO_2_ must cross the alveolar epithelium, the basal lamina, and the endothelial cells that form the capillaries. Therefore, these layers are referred to as the alveolar-capillary barrier. This barrier has a distance of less than 1 *μ*m, a diffusion distance that is thin enough to allow efficient gas exchange. Thus, the anatomy of the mammalian lung and the structure of the alveoli satisfy Fick's law of diffusion in terms of requirements for a large surface area and thin diffusion distance and establish the physical requirements for optimal gas exchange of air breathing mammals. 

The consequence of the proximity of the capillaries to the alveolar epithelium, however, is that small amounts of liquid are permanently forced into the alveolar airspaces due to blood pressure. This fluid contributes to alveolar lining fluid, facilitating diffusion of dissolved gases such as O_2_ and CO_2_. However, increased fluid volume in the alveoli characterised leads to an extension of the gas diffusion distance. Therefore, mechanisms must exist which remove infiltrated fluid from the alveoli—a process referred to as *alveolar fluid clearance*. For this, the reabsorption of Na^+^ from the alveoli, especially via the activity of Na^+^ channels in the pulmonary epithelium, is of particular importance and will be discussed in further detail below.

## 2. Na^+^ Channels and Their Role in Alveolar Fluid Clearance

**Figure 1:**
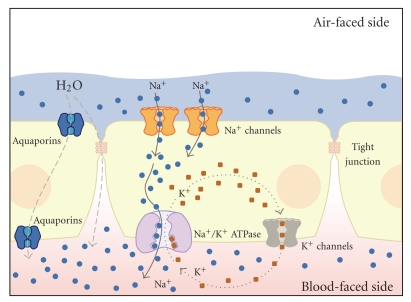
Transepithelial Na^+^ transport drives alveolar fluid clearance. Na^+^ enters the cell interior passively following an electrochemical gradient via Na^+^ channels, which are located at the apical membrane of alveolar epithelial cells. The Na^+^ ions are then actively pumped out of the cells by the Na^+^/K^+^-ATPase in exchange for K^+^ ions, which leave the cell afterwards via basolaterally localized potassium channels. Thus, there is a net movement of Na^+^ ions from the apical (air-faced) to the basolateral (blood/interstitium-faced) side of the alveolar epithelium. This creates osmotic forces, and, consequently, water follows out of the airspaces across the epithelium either paracellularly via tight junctions or transcellularly via aquaporins. The figure has been modified from [2].

As described above, AT1 and AT2 cells are linked together by tight junctions. The formation of tight junctions between these epithelial cells not only results in a tight linkage of the cells to one another, but also limits the free diffusion of transmembrane protein complexes. Thus, the protein repertoire of the apical membrane of the alveolar epithelium differs from that of the basolateral membrane. This issue is of particular importance for transepithelial Na^+^ transport, a mechanism that is crucial for alveolar fluid clearance. Transepithelial Na^+^ transport occurs primarily through the interplay of two transport systems: Na^+^permeable ion channels, such as the epithelial Na^+^ channel (ENaC), located at the apical membrane, as well as the basolaterally localized Na^+^/K^+^-ATPase (Figure 1). The Na^+^ ions enter the cells, following an electrochemical gradient, at the apical membrane via Na^+^ channels and are extruded at the basolateral side by the activity of the Na^+^/K^+^-ATPase. This leads to a net movement of Na^+^ from the apical to the basolateral side of the alveolar epithelium. This transepithelial Na^+^ transport in turn creates osmotic forces which drive the movement of water from the apical to the basolateral side. Water crosses the alveolar epithelium either paracellularly via tight junctions or transcellularly via water channels, or *aquaporins*, which are expressed in alveolar type 1 cells [1]. Water is eventually removed from the lungs via the lymphatic or capillary system. 

Thus, alveolar fluid clearance is a direct consequence of transepithelial ion and, particularly, Na^+^ transport (Figure 1). This correlation was demonstrated in a study by Hummler et al., where knock-out mice that did not express the alpha subunit of the epithelial Na^+^ channel (ENaC) in the alveolar epithelium died after birth due to defective neonatal fluid clearance and fluid accumulation in the lungs [3]. Consistent with this study, lung-specific knockdown of *α*ENaC using siRNA decreased baseline fluid clearance in rats *in vivo* [4]. 

These examples underline the fact that Na^+^ channels in the pulmonary epithelium play a key role in driving alveolar fluid clearance and, thus, the regulation of the fluid content of the airspaces in the lung.

## 3. Na^+^ Channels in the Pulmonary Epithelium

Several types of Na^+^ channels have been described in alveolar epithelial cells, including channels sensitive to the diuretic *amiloride* as well as cyclic nucleotide gated cation channels [5, 6]. Amiloride-sensitive Na^+^ channels in particular are thought to represent the major pathway for apical Na^+^ entry into alveolar epithelial cells [7, 8]. Their contribution to alveolar fluid clearance has been demonstrated in studies, which show that amiloride is able to block active Na^+^ transport and fluid clearance in isolated lung models [9–13] and *in vivo *in animal studies. This finding was recently confirmed by using genetically engineered mice with mutations conferring hypo- or hyperactivity of the amiloride-sensitive epithelial sodium channel (ENaC). Those studies demonstrated that the fluid content of the lungs is highly dependent on the activity of amiloride-sensitive Na^+^ channels, thus illustrating the major contribution of these channels to alveolar fluid clearance [14–16].

Two distinct types of amiloride-sensitive Na^+^ channels have been described in alveolar epithelial cells: highly selective Na^+^ channels (HSCs), which are characterized by a high selectivity towards Na^+^, and nonselective cation channels (NSCs), with no selectivity for Na^+^ over K^+^ [5, 6, 17–19]. The HSCs are also referred to as “epithelial Na^+^ channel (ENaC)-like” Na^+^ channels [20]. The classical ENaC consists of three subunits, *α*, *β* and *γ*  [21], which might assemble as a heterotrimer to build a Na^+^-permeable channel spanning the cell membrane [22]. When these three subunits of ENaC are coexpressed in *Xenopus laevis* oocytes, the resulting expressed channel has almost identical characteristics to the HSCs identified from lung cells [18, 23, 24]. Therefore, the HSC observed in lung epithelia might be ENaC consisting of the *α*, *β* and *γ* subunits [6, 18, 25, 26]. In contrast to the HSCs, the structure and subunit composition of NSCs are still not completely understood [19, 20]. It is speculated that NSCs might solely be formed by the *α* subunit of ENaC [27]. In order to clarify these topics, it is important to identify the precise structure and subunit stoichiometry in which the classical ENaC subunits might assemble to form either selective or nonselective ion channels. In this regard, it is also noteworthy that an additional subunit of ENaC (*δ*) has been described in humans [28] which has at least two functionally different splice isoforms [29, 30] that are also expressed in the human lung (unpublished observations) and lung epithelial cells [31, 32]. The *δ* subunit of ENaC can replace the *α* subunit, forming Na^+^ channels together with *β* and *γ* subunits when heterologously expressed in *Xenopus laevis* oocytes [28]. Interestingly, these channels have different biophysical properties when compared to channels containing the *α* subunit [31]. To what extent, the  *δ* ENaC subunits might be involved in, for example, forming NSCs and how these subunits might play a role in amiloride-sensitive pulmonary transepithelial Na^+^ transport and alveolar fluid clearance however, remain to be elucidated. 

It should be mentioned that there is also a fraction of active Na^+^ transport and alveolar fluid clearance which is insensitive to amiloride [33]. However, which Na^+^ channels or Na^+^-coupled transporters are involved in amiloride-insensitive transport across the alveolar epithelium remains unknown (for detailed review see [33]).

Aside from the identification of the precise structure and composition of Na^+^ channels in the distal lung, an important aspect is the question as to where in the distal lung these channels are expressed. As described earlier, the alveolar epithelium consists of two cell types: AT1 and AT2 cells. Although AT1 cells represent less than 10% of the cells in the lung, they form more than 98% of the lung surface area [1]. In this regard, the classical paradigm was that AT1 cells are “biologically inert” cells that just contribute to the thin alveolar-capillary barrier whereas the “biologically active” cells are AT2 cells [1]. Following that paradigm, until recently, the general view was that transepithelial Na^+^ absorption takes place solely by AT2 cells. However, given that AT2 cells correspond to less than 2% of the total lung surface area, it seems surprising that these cells alone manage to drive alveolar fluid clearance by transepithelial Na^+^-transport. The idea of AT2 cells as the Na^+^ transporting cell in the distal lung is largely rooted in the fact that these cells have been experimentally approachable for more than 30 years [34] and have been intensively investigated. In contrast, techniques to isolate and investigate pure populations of AT1 cells have only recently been developed [1, 35, 36]. Since then, there have been experimental hints that isolated AT1 cells also express Na^+^ channels (HSC and NSC) [8, 37, 38]. It should be mentioned that studies with AT2 cells have shown that the expression of Na^+^ channels in isolated alveolar cells is highly sensitive to culture conditions [39], and this makes it difficult to integrate data from isolated cells into a physiological context. However, more recent data from the Eaton group delivered hints for the existence of HSCs and NSCs in AT1 cells of lung slice preparations [40, 41]. Thus, it seems that AT1 and AT2 cells both express the Na^+^ channel repertoire which makes them suitable for transepithelial Na^+^ transport. Therefore, the classical paradigm of pure AT2-driven Na^+^ absorption changes into the view that Na^+^ absorption takes place across the entire alveolar epithelium mediated by Na^+^ channels which are expressed both in AT1 and AT2 cells.

## 4. Na^+^ Channels and the Development of Pulmonary Edema

**Figure 2:**
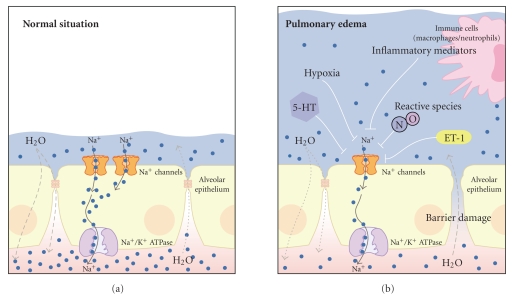
Impaired Na^+^ channel activity is associated with the development of pulmonary edema. (a) under normal conditions transepithelial Na^+^ transport mediated via Na^+^ channels in the alveolar epithelium drives water reabsorption from the airspaces to the interstitium. This mechanism counteracts water filtration into the airspaces and keeps the fluid layer covering the alveolar epithelium low. (b) a variety of factors that inhibit Na^+^ channels in the alveolar epithelium have been identified under diseases associated with pulmonary edema such as HAPE or ALI/ARDS: hypoxia, inflammatory mediators which are released by activated immune cells (such as macrophages or neutrophils), endothelin 1 (ET-1), reactive species such as nitric oxide (NO), or factors which are released due to hypoxia or epithelial stress such as serotonin (5-HT). The decreased activity of Na^+^ channels leads to decreased water reabsorption and fluid accumulation in the airspaces. Under pathological conditions such as HAPE or ALI/ARDS, there is additionally increased fluid filtration into the airspaces due to impaired epithelial barrier integrity. The consequence of both impaired Na^+^ and thus water reabsorption and increased fluid filtration is the development of pulmonary edema. The figure has been modified from [2]. For clarity, aquaporins and potassium fluxes/channels, as indicated in Figure 1, have been omitted.

Although many open questions remain concerning the precise Na^+^ channel structure and spatial expression, the correlation between Na^+^ channel activity in AT1 and AT2 cells and alveolar fluid clearance implies that there may be a link between dysregulated Na^+^ channel activity and the development of pulmonary edema due to impaired resolution of fluid (Figure 2). Evidence for this assumption comes from transgenic mice with loss-of-function mutations of amiloride-sensitive HSCs [14, 16]. These studies demonstrated that hypoactive Na^+^ channels in the lung not only lead to impaired alveolar fluid clearance but also are a predisposing factor for the development of pulmonary edema [14].

The physiological importance of this association is evident when one considers the development of high-altitude pulmonary edema (HAPE). Mountaineers at high altitude are faced with physical problems: decreased atmospheric pressure, hypoxia, and pulmonary hypertension due to hypoxic vasoconstriction. The lower atmospheric pressure leads to an increased pressure gradient between the airspaces of the lung and the body interior. Thus, the described leakage of fluid—as a result of blood pressure—is enhanced. In addition, pulmonary blood vessels respond to hypoxia with vasoconstriction, a mechanism that usually prevents nonventilated alveoli from being perfused. The pulmonary blood pressure further increases fluid filtration into the lungs. In addition, there is also an impairment of barrier integrity under HAPE which augments alveolar flooding (as reviewed in [42]). The situation becomes even more problematic, since there is also an impairment of fluid resolution due to impaired Na^+^-transport, and the activity of Na^+^ channels in particular. This phenomenon is *inter alia* due to hypoxia-induced inhibition of Na^+^ channels [43, 44]. The decreased Na^+^ channel activity in hypoxic lungs is likely due to hypoxia-induced Na^+^ channel retrieval from the alveolar epithelial cell surface without affecting total expression of Na^+^ channels in the lung [45]. However, experimental studies concerning the latter issue delivered controversial results [45–48], which might be due to the different degrees of hypoxia employed in the used models. Nevertheless, the overall effect of hypoxia is an impaired transepithelial Na^+^ transport, which is—at least in part—due to impaired Na^+^ channel activity in the alveolar epithelium. This eventually leads to a reduction of fluid reabsorption from the alveoli and thus contributes to the development of pulmonary edema.

Taken together, the conditions leading to HAPE demonstrate how pulmonary edema can develop as a combination of both increased fluid filtration and impairment of transepithelial Na^+^ transport, especially epithelial Na^+^ channel activity (Figure 2). In this regard, there is also an association between transepithelial Na^+^ transport and a human lung disease which is referred to as *acute lung injury *(ALI) or *acute respiratory distress syndrome* (ARDS [49]). Apart from pronounced inflammation and epithelial damage, pulmonary edema is a hallmark of this disease [50, 51]. The formation of pulmonary edema in ALI/ARDS occurs due to damage to the alveolar-capillary barrier, which leads to fluid leakage into the alveoli and also due to defective alveolar fluid clearance mechanisms [49]. Thus, in addition to increased edema formation due to epithelial damage, there is also an impairment of the resolution of edema due to diminished alveolar fluid clearance, which is dependent on the efficacy of transepithelial Na^+^ transport [51]. There is a correlation between transepithelial Na^+^ transport and edema clearance in ALI/ARDS patients: patients that have a functional transepithelial Na^+^ transport exhibit improved pulmonary edema resolution and have a better clinical outcome compared to patients with defective transepithelial Na^+^ transport [51]. 

Thus, in the described pathophysiological situations, HAPE and ALI/ARDS, there is a link between transepithelial Na^+^ transport, Na^+^ channels in particular, and the development of pulmonary edema. Following that line, a variety of factors have been identified which might account for a decreased activity of Na^+^ channels under these pathophysiological conditions (Figure 2). Increased synthesis of nitric oxide (NO) due to, for example, upregulation of nitric oxide synthases, has been demonstrated in ALI/ARDS [52, 53]. Furthermore, NO decreased the activity of Na^+^ channels (HSC and NSC) in alveolar epithelial cells [23, 41, 54, 55]. Thus, there might be a link between the development or persistence of edema in ALI/ARDS and NO-mediated inhibition of Na^+^ channels. By contrast, defective NO synthesis is observed under HAPE [42]. However, this putatively beneficial effect with respect to Na^+^ channel activity might be outweighed by exaggerated pulmonary hypertension and thus increased fluid filtration into the alveoli [42].

Another factor which might account for impaired Na^+^ channel activity and pulmonary edema is endothelin 1 (ET-1). ET-1 is a vasoconstrictor which regulates pulmonary vascular tone [56]. Increased levels of ET-1 have been demonstrated in HAPE [57] and ALI/ARDS [58]. In addition, ET-1 inhibits epithelial Na^+^ channels *in vitro* [59] and decreases alveolar fluid clearance in rats [60]. Thus, ET-1 not only leads to enhanced fluid filtration due to pulmonary hypertension, but might also represent a key factor that impairs the activity of Na^+^ channels and thus impairs the resolution of pulmonary edema in patients with HAPE or ALI/ARDS. 

Both examples, NO and ET-1, demonstrate how dysregulated Na^+^ channel activity might occur under conditions as HAPE or ALI/ARDS. Apart from ET-1 and NO, a variety of other factors have been identified which may also contribute to a decreased activity of Na^+^ channels and hence edema development (Figure 2): inflammatory mediators such as interleukin-1beta [61] or tumor necrosis factor-alpha [62] or factors, such as serotonin, which are released as a result of hypoxia or epithelial stress [63].

Thus, apart from hypoxia, there are intrinsic factors that occur in diseases associated with pulmonary edema which might contribute to disturbed fluid clearance, and hence edema resolution, by interference with Na^+^ channels in the alveolar epithelium (Figure 2). Therefore, Na^+^ channels can be regarded as key players with respect to edema formation and might be promising targets for the treatment of pulmonary edema.

## 5. Na^+^ Channels as Molecular Targets for the Treatment of Pulmonary Edema?

The described examples, HAPE and ALI/ARDS, demonstrate that pathological situations in the lung which are associated with pulmonary edema can be correlated with an impaired activity of Na^+^ channels and transepithelial Na^+^ transport. Consequently, it might be questioned whether enhancement of Na^+^ channel activity would enhance edema resolution and improve the clinical outcome of patients with pulmonary edema.

Experimental evidence that enhanced Na^+^ channel activity might indeed improve edema resolution comes from studies using transgenic mice with hyperactive Na^+^ channels [64]. These mice carry a mutation in the *β*-subunit of the epithelial Na^+^ channel, ENaC, which leads to impaired channel retrieval, and thus, persistence of ENaC at the cell surface [65, 66]. This mutation is the genetic reason for a hereditary form of hypertension referred to as Liddle's syndrome [67]. Consistent with the association of Na^+^ channel activity and alveolar fluid clearance, baseline fluid clearance was increased in mice carrying the *β*-Liddle mutation compared to wild types [64]. Moreover, these mice were able to resolute hydrostatic pulmonary edema (induced by volume overload due to saline infusion) much better than wild-type mice [64]. These results demonstrate that increasing Na^+^ channel activity might be a putative tool to potentiate alveolar fluid clearance and thereby enhance the resolution of pulmonary edema. 

In this regard, *β*-adrenergic agonists are prominent activators of Na^+^ channels in the alveolar epithelium and therefore stimulators of alveolar fluid clearance (for detailed review see [7]). This finding has been confirmed recently in mutant mice with low expression of epithelial Na^+^ channels (*β*-ENaC) which show no increase in alveolar fluid clearance upon *β*-agonist treatment [16].

Consistent with the idea of *β*-agonists as potential therapeutic tools, *β*-adrenergic agonist treatment improved fluid clearance and edema resolution in experimental models of ALI/ARDS [68–71]. The activation of transepithelial Na^+^ transport and alveolar fluid clearance by *β*-adrenergic agonists was also shown to reduce extravascular lung water in patients who were part of the so-called BALTI trial (*beta-agonist lung injury trial, BALTI*), a clinical trial that addressed the possibility of *β*-agonist treatment in ALI/ARDS [72]. Recent data also suggest that *β*-agonist treatment might restore Na^+^ absorption and epithelial Na^+^ channel activity to normal levels in hypoxic alveolar epithelial cells from rats [73]. Consistently, *β*-adrenergic agonist inhalation reduced the incidence of HAPE likely by stimulated fluid absorption [74]. 

The described studies with *β*-adrenergic agonists demonstrate that activation of Na^+^ transport, *inter alia* by stimulating Na^+^ channels, might indeed be a promising strategy to improve edema resolution. Thus, Na^+^ channels might indeed represent molecular targets for the treatment of pulmonary edema. However, it is important to note that in diseases like HAPE or ALI/ARDS there are three steps that account for the development of pulmonary edema: (i) alveolar flooding due to increased fluid filtration (ii) disturbances in the epithelial barrier integrity, and (iii) impaired fluid clearance due to impaired transepithelial Na^+^ transport. Although there are experimental studies demonstrating that edema formation can be the result of diminished Na^+^ transport despite of an intact epithelial barrier [75], especially edema resolution driven by transepithelial Na^+^ transport can only take place over an intact epithelial barrier. Barrier leakage is beside impaired Na^+^ transport a characteristic of HAPE [76] or ALI/ARDS [7]. Whereas damage to the alveolar epithelium is the major cause of barrier disruption in patients with ALI/ARDS, it is speculated that an impairment of barrier integrity under HAPE—independently of Na^+^ channel activity—might be the result of enhanced leakiness of alveolar epithelial tight junctions [77]. Independent of its cause, barrier damage is an important factor that has to be carefully taken into consideration [78]. Therefore, Na^+^ channels cannot be the only molecular target for putative therapeutic tools regarding the treatment of pulmonary edema under conditions such as HAPE or ALI/ARDS. Reducing the cause of fluid filtration into the alveoli and restoring especially the epithelial barrier integrity has to be a prerequisite for enhanced Na^+^ transport to be effective for edema resolution.

This correlation has already been implicated in the BALTI trial [72]. The reduction of extravascular lung water by *β*-adrenergic agonist treatment was only apparent 72 h after the beginning of treatment [72]. This observation might demonstrate that for effective edema resolution to take place, an improvement in barrier integrity is necessary. Following that line, there are interesting data suggesting a role of *β*-adrenergic agonists in stimulating barrier integrity *in vitro* [79] and in patients with ALI/ARDS [80].

Therefore, future therapeutic strategies to improve edema resolution must focus on (i) a reduction of alveolar flooding, for example, by reduction of hypoxia and pulmonary hypertension (ii) restoration of barrier integrity and finally (iii) enhancement of transepithelial Na^+^ transport, for example, by stimulating Na^+^ channels in the alveolar epithelium.

## 6. Concluding Remarks

Taken together, Na^+^ channels in alveolar epithelial cells represent important mediators of alveolar fluid clearance. Understanding the precise structure and regulation of Na^+^ channels under physiological conditions in the lung as well as their dysregulation under pathological conditions such as HAPE or ALI/ARDS is a prerequisite for understanding the pathogenesis of lung diseases associated with pulmonary edema and the development of new therapeutic strategies. It is important to point out that Na^+^ transport mediated alveolar fluid clearance can only take place across an intact epithelial barrier. Thus, the challenge of future therapeutic approaches to treat pulmonary edema will be to minimize edema formation due to barrier damage or increased filtration and to enhance edema resolution by stimulating Na^+^ transport and, particularly, Na^+^ channels in the alveolar epithelium.
